# THE EFFECTS OF EXERCISE ON BRAIN FUNCTIONAL CONNECTIVITY IN ADULTS AGED 55–65 EXPOSED TO EXPERIMENTAL BED REST

**DOI:** 10.1093/geroni/igae098.3495

**Published:** 2024-12-31

**Authors:** Guilherme Balbim, Chun-Liang Hsu, Daria Tai, Simon Duchesne, Kenneth Madden, Teresa Liu-Ambrose

**Affiliations:** University of British Columbia, Vancouver, British Columbia, Canada; The Hong Kong Polytechnic University, Hong Kong, Hong Kong; University of British Columbia, Vancouver, British Columbia, Canada; Université Laval, Quebec, Quebec, Canada; University of British Columbia, Vancouver, British Columbia, Canada; University of British Columbia, Vancouver, British Columbia, Canada

## Abstract

Bed rest can occur because of chronic conditions, injury, or hospitalization and initiate a downward spiral of deleterious changes in several body systems. Exercise is a countermeasure to many of these changes. However, its impact on middle-aged and older adults’ brain functional connectivity (FC) during bed rest remains unexplored. We examined the effects of exercise on brain resting-state FC in adults exposed to 14 days of experimental 6° head-down bed rest (HDBR). We conducted a non-blinded, parallel-group, randomized controlled trial with 23 healthy adults aged 55-65. Participants were randomized to either 14 days of HDBR (CON) or 14 days of HDBR with daily aerobic and strength exercise training (EX). At baseline and HDBR completion, participants underwent brain structural and resting-state functional magnetic resonance (MRI). Resting-state MRI preprocessing was performed using the FMRIPREP version 20.2.3. We extracted preprocessed resting-state time-series from a priori networks of interest based on Yeo’s 7-network parcellation. We conducted complete-case data analysis (NEX=11; NCON=8). Analysis of covariance explored EX effects on resting-state FC after controlling for baseline resting-state FC and baseline physical activity assessed by the Physical Activity Scale for the Elderly. Changes in EX resting-state FC within- and between- the Visual, Sensorimotor, Dorsal Attention, Ventral Attention, Fronto-Executive, Frontoparietal, and Default Mode Networks were not statistically significant compared with CON (all ps > 0.05). Exposure to 14 days of experimental HDBR or 14 days of daily aerobic and strength exercise training in HDBR did not elicit changes in brain resting-state FC of healthy middle-aged and older adults.

